# Draft Genome Sequence of Methicillin-Sensitive *Staphylococcus aureus* ATCC 29213

**DOI:** 10.1128/genomeA.01095-15

**Published:** 2015-09-24

**Authors:** Isha Soni, Harinath Chakrapani, Sidharth Chopra

**Affiliations:** a Division of Microbiology, CSIR-Central Drug Research Institute, UP, Lucknow, India; b Department of Chemistry, IISER Pune, Maharashtra, India

## Abstract

*Staphylococcus aureus* subsp. *aureus* ATCC 29213 is one of the most commonly used strains in drug discovery research and for quality control. We report the completed draft genome sequence for the strain.

## GENOME ANNOUNCEMENT

*Staphylococcus aureus* is an opportunistic human nosocomial and community-associated pathogen that causes a wide spectrum of infections ranging from skin and skin structure infections to bacteremia and necrotizing pneumonia (1). It has developed into a successful pathogen, owing to its numerous virulence factors and its resistance to a multitude of antibiotics, which narrows the options available for its treatment. Recently, a number of genomic efforts have been directed toward understanding the genetic basis of antimicrobial resistance and virulence by comparing the genomes of drug-resistant strains to those of drug-susceptible strains (2, 3). This has been hampered, however, by the unavailability of genomes of a number of drug-susceptible strains, especially those that are methicillin-susceptible, and thus our quest to determine the genome sequence of *Staphylococcus aureus* ATCC 29213.

*Staphylococcus aureus* subsp. *aureus* ATCC 29213 is a clinical isolate with the designation Wichita that is utilized as a standard quality-control strain in laboratory testing. It is sensitive to a large variety of antimicrobials, including methicillin. *Staphylococcus aureus* subsp. *aureus* ATCC 29213 was purchased from American Type Culture Collection (ATCC) and was grown overnight at 37°C in Mueller-Hinton broth (Becton, Dickinson) and genomic DNA was extracted using QiAmp DNA minikit (Qiagen) according to the manufacturer’s instructions.

The whole genome was sequenced on the Ion Torrent PGM system (Life Technologies) following the manufacturer’s protocols for 200-bp genomic DNA (gDNA) fragment library preparation (Ion Xpress Plus gDNA and Amplicon Library Preparation), template preparation (Ion OneTouch system), and sequencing (Ion PGM 200 sequencing kit). A total of 363 Mb of data were obtained with an accumulated length of 320,957,160 bp (115× fold coverage). A total of 147 contigs were assembled using Assembler SPAdes version 4.4.0.1 to give a genome length of 2,773,559 bp with an average GC content of 34%. The annotation was carried out using RAST version 2.0 (http://rast.nmpdr.org; [4]). A total of 2,907 open reading frames were predicted, out of which 780 (26.8%) had an assigned function and 32 (1.1%) were conserved hypothetical, while the remaining 812 (27.9%) were classified as either of unknown function and/or hypothetical proteins with 53 tRNAs as well as 9 rRNAs. The detailed genomic data analysis from this *de novo* genome assembly is under way to obtain a finer resolution.

Overall, the availability of the genome sequence of methicillin-sensitive *S. aureus* ATCC 29213 facilitates additional comparative genomic and bioinformatics analysis of drug-resistance mechanisms in *S. aureus.*

### Nucleotide sequence accession number.

This whole-genome project has been deposited in DDBJ/EMBL/Genbank under the accession number LHUS00000000.
